# Water environmental stress, rebound effect, and economic growth of China’s textile industry

**DOI:** 10.7717/peerj.5112

**Published:** 2018-06-29

**Authors:** Yi Li, Jie Shen, Linyi Lu, Yan Luo, Laili Wang, Manhong Shen

**Affiliations:** 1 Fashion School, Zhejiang Sci-Tech University, Hangzhou, China; 2 Clothing Engineering Research Center of Zhejiang Province, Zhejiang Sci-Tech University, Hangzhou, China; 3 Silk and Fashion Culture Research Center of Zhejiang Province, Zhejiang Sci-Tech University, Hangzhou, China; 4 School of Economics and Management, Zhejiang Sci-Tech University, Hangzhou, China; 5 Fashion School, Donghua University, Shanghai, China; 6 School of Business, Ningbo University, Ningbo, China

**Keywords:** Textile industry, Wastewater discharge, Water environmental stress, Rebound effect, COD discharge, Economic growth, Decoupling

## Abstract

The rapid development of China’s textile industry (TI) has led to severe water environmental stress. Water environmental stress of China’s TI mainly comes from large quantities of discharged wastewater and chemical oxygen demand (COD). The sustainable development of the TI is realized to achieve the decoupling between economic growth and water environmental stress. This study analyzes the decoupling elasticity results from wastewater discharge and COD discharge, respectively. Decoupling results show that TI’s wastewater has strong decoupling from economic growth for three years (2002, 2013–2014) while COD has strong decoupling for six years (2002–2003, 2008, 2010, 2013–2014). The paper further calculates the decoupling elasticity results of the TI’s three sub-sectors (manufacture of textile sector, manufacture of textile wearing and apparel sector, and manufacture of chemical fibers (MCF) sector), and calculates the factors that affect wastewater discharge. The decrement and rebound effects of wastewater discharge are analyzed based on calculated results. Decomposition results show that the scale factor is the most significant contributor to wastewater discharge, the intensity factor inhibits wastewater discharge, and the effect of the structure factor is not evident. The decrement effect of TI increases yearly, but the rebound effect shows that the absolute amount of wastewater discharge also increases. The rebound effect has declined since 2012. In the three sub-sectors, MCF’s decrement effect is the strongest, and its rebound effect is the weakest, which indicate that MCF is the biggest contributor to the discharge reduction of China’s TI.

## Introduction

The textile industry (TI), one of the traditional pillar industries in China, has greatly contributed to economic growth, export earnings, and employment promotions. China’s TI total output value increased from USD 31.36 billion in 1997 to USD 27.43 trillion in 2014, with an average annual growth rate of 10.96% (based on the 2014 price) and a serious wastewater and pollutant discharge problem. From 1997 to 2014, the TI’s wastewater discharge increased from 1,571.11 to 2,537.68 Mt, with an average annual growth rate of 2.70% (Ministry of Environmental Protection of the People’s Republic of China, 1998–2006, 2006–2014). The TI is traditionally one of the highest polluting industries in China. The main pollutant in the TI’s wastewater, chemical oxygen demand (COD), has ranked fourth among China’s 41 key survey industries for 12 years (2003–2014). The TI’s wastewater discharge ranked third among China’s 41 key survey industries for four consecutive years (2011–2014).

The textile industry’s wastewater is produced mainly by the printing and dyeing industry and the chemical fiber industry; water pollutants from the printing and dyeing industries are serious (Yu et al., 2005). In Shaoxing City, China, where printing and dyeing industries are highly concentrated, the average daily discharge of printing and dyeing wastewater exceeds 0.60 Mt, which accounts for 83.14% of Shaoxing’s total industrial wastewater discharge (Liu et al., 2016). Water pollution also causes severe groundwater contamination and reduces seafood production, thus imposing heavy burden on local social stability. Government attaches great importance to the TI’s water pollution. In response to the industry growth and sustainable development goals in China’s “13th Five-Year Plan” (Ministry of Industry and Information Technology of the People’s Republic of China, 2016b), and in order to achieve the decoupling of TI’s economic growth and water environmental stress and develop in a highly efficient and sustainable way, TI needs to break through the original production model, change its high pollution production characteristics and reduce water environmental stress. Decoupling describes a state of relations of gradual decline or even separation among mutually related variables. The Organization for Economic Co-operation and Development (OECD) defines decoupling as a break of relationships between environmental stress and economic growth (Organisation for Economic Co-Operation and Development (OECD), 2001).

Based on this definition, the present research clarifies decoupling as the independent relation hidden between water environmental stress and economic growth. Water environmental stress should involve the consideration of both the quality and quantity of wastewater (Pang & Abdullah, 2013). The COD is a typical and key pollutant in TI’s wastewater, so it is another index of water environmental stress. Excessive COD content will lead to hypoxia and death of aquatic organisms, and the water will become rotten (Wang, Luan & Kang, 2013). Decoupling degree is evaluated by the decoupling index. OECD decoupling indicators (OECD, 2002), and Tapio decoupling indicators (Tapio, 2005; Tapio et al., 2007) are two of the most widely accepted decoupling indices. The latter classification further breaks down the index into eight categories based on the former, which is strong decoupling, weak decoupling, recessive decoupling, strong negative decoupling, weak negative decoupling, expansive negative decoupling, expansive coupling, and recessive coupling.

Decoupling theory is widely used in evaluating the relationship between resources, environment, and economic growth. Sorrell et al. (2012) applied the decoupling model to analyzing the decoupling relationship between Britain road transport energy consumption and GDP between 1989 and 2004. They argued that the decline in commodity prices relative to GDP was the main reason for relative decoupling. Akulov (2014) conducted a case study of the Kemerow state in Russia when studying the ecological impact of coal mining and found that an increase in coal production intensified regional water pollution; thus, decoupling was not found in the area. Van Caneghem et al. (2010) studied the eco-efficiency of the Flemish industry in Belgium and found that energy consumption and waste emissions increased as yields increased. However, based on ecological indicators, the pressure on economic growth was reduced and economic growth and ecological benefits achieved relative decoupling. Conrad & Cassar (2014) analyzed Malta’s (a single small island country) economic and environmental data during the study of the decoupling relationship and concluded that selecting individual environmental variables may vary the decoupling result. Wu & Wang (2013) chose industrial wastewater discharge and COD content in wastewater as water environmental stress indicators, built a decoupling index system, and used it to verify China’s economic growth and wastewater discharge decoupling status between 1986 and 2010. Guo & Zhang (2013) fit wastewater and COD discharge into pollutants index and calculated the decoupling degree, the results showed that in the study years, COD discharge of China’s industry was decreasing and the proportion of industrial COD discharge decreased gradually, indicating that reduction effect existed in China’s industrial COD discharge. Lei, Lei & Xu (2014) used the IPAT model to construct the decoupling model of economic development and wastewater discharge and verified the example of Wuhan City in China. The relationship between economic development and wastewater discharge in Wuhan was already decoupled, but the ideal decoupling state has not been achieved. Li & Cao (2014) decomposed the decoupling index into scale decoupling elasticity, technical decoupling elasticity, and structural decoupling elasticity and analyzed the mechanism of the relationship between economic development and environmental pollution in China from 1990 to 2011; they found that the decoupling degree of China’s economic situation was highly relevant to macro-control policies. Wang (2013) calculated the gray water footprint of China’s TI, used the decoupling index to analyze the decoupling status between gray water footprint and economic growth, and found that the gray water footprint has a strong decoupling development trend. Gilmont (2015) calculated the water footprint of the food industry in the Middle East and North Africa countries from 1961 to 2009, decoupled the water footprint of the social development and food industry, and believed that increasing the ratio of imported food in food consumption in the country could help reduce the blue water footprint. Li et al. (2017) considered water resources and water environment as a whole, used the water footprint to quantify water resources and water environment, studied the decoupling relationship between economic growth and water footprint in China’s TI, and found that the TI’s development has not completely dissociated from the water footprint.

The amount of wastewater and COD discharged from unit economic output must be controlled and reduced to realize the “strong decoupling” between economic growth and water environmental stress brought by TI. Decrement and rebound effects are introduced, and the environmental impact cancellation caused by the enlargement of the economic scale and the increase of efficiency on the basis of factor decomposition is considered (Matiaske, Menges & Spiess, 2012). It is conducive to evaluate the current situation of China’s TI emission reduction objectively and comprehensively, and some pertinent suggestions are proposed to realize an ideal decoupling status.

The paper is organized as follows. The “Methodology and Data” is the main method and data source for the study of economic growth and water environmental stress in the TI. “Results and Discussion” evaluates the wastewater discharge of the TI and its three sub-sectors, discusses and analyzes their decoupling calculation results, and sums up the main results of the decomposition analysis. The final section presents the “Conclusion”.

## Methodology and Data

### Decoupling method

The decoupling elasticity between water environmental stress and economic growth of TI can be expressed as follows:

(1) $$D{\rm{ \,=\, }}{{{\rm{\% }}\Delta W} \over {{\rm{\% }}\Delta P}}{\rm{ = }}{{\left( {{W^T} - {W^{T - 1}}} \right)/{W^{T - 1}}} \over {\left( {{P^T} - {P^{T - 1}}} \right)/{P^{T - 1}}}}{\rm{ = }}{{{W^T}/{W^{T - 1}} - 1} \over {{P^T}/{P^{T - 1}} - 1}}$$

In Eq. (1), *D* is the decoupling elasticity of wastewater discharge, *W* is the water environmental stress from TI, *W^T^*, and *W^T^*^−1^ are the discharge volumes for years *T* and *T*−1, respectively, *P* is the economical indicator, and *P^T^* and *P^T^*^−1^ are the output values for years *T* and *T*−1, respectively. Thus, the decoupling elasticity *D* is determined by using the ratio of change rate between water environmental stress (%Δ*W*) and industrial output value (%Δ*P*). The calculation of decoupling elasticity is a non-dimensional process.

**Figure 1:**
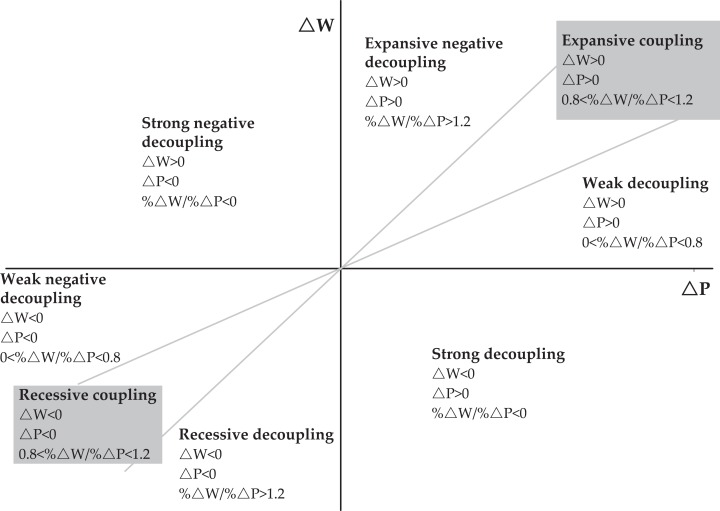
Decoupling degree of economic growth and water environmental stress.

The decoupling relationship under the elastic index method can be further sub-divided into eight levels based on the relationship between decoupling and coupling (Tapio, 2005). Changes in water environmental stress and economic growth can be divided into coupling, decoupling, and negative decoupling. A more detailed segmentation falls within the range of ±20% of $\% \Delta W/\% \Delta P\,{\rm{ = }}\,{\rm{1}}$, which is still regarded as elastic coupling. The positive or negative growth from the variable itself is expressed as an expansion of coupling and recessive coupling. A concrete classification of decoupling degree is shown in Fig. 1. According to the definition, strong decoupling represents the most favorable decoupling state and strong negative decoupling represents the least favorable decoupling state. Under the condition of weak decoupling, the economic growth rate is still faster than the increasing speed of water environmental stress, so it also indicates a relative good decoupling status as compared with other states.

### LMDI decomposition method

The decomposition method is an analytic procedure to explore the potential structure of target variables and study the mechanism of object action. Index decomposition analysis (IDA) is one of the main research methods to break down influencing factors. The basic idea of the IDA method is to upward trace the change of the target variable, decompose the variable into several combinations of influencing factors, and analyze the contribution degree of different factors to determine highly contributing factors objectively (Xu & Liu, 2014).

The Divisia index method introduced by Divisia is one of the most commonly used exponential decomposition methods. Hulten (1973) elaborated and described the method in detail and applied the exponential decomposition method for the study of energy issues. The multiplication and addition forms of the arithmetic mean of the Divisia index method has been proposed (Boyd et al., 1987; Boyd, Hanson & Sterner, 1988). Ang & Pandiyan (1997) presented a modified Divisia decomposition method, the logarithmic mean Divisia exponential decomposition method (LMDI), which eliminated the residual terms in the decomposition to avoid the arbitrariness of parameter estimation and enhance the credibility of the results. A logarithmic operation could not calculate zero and negative values (Ang & Liu, 2007; Ang & Zhang, 2000), making the Divisia decomposition method can be used for any case analysis.

The LMDI decomposition method is widely used in the analysis of influencing factors, and the multiplication and addition forms of LMDI can be converted by formulas. Compared with multiplication decomposition, it is easier to analyze the decomposition results through addition decomposition. Therefore, the addition form is used to construct the decomposition model. The equation is expressed as follows:

(2) $$W{\rm{\,=\,}}\sum\limits_{i = 1} {\left({{{{W_i}} \over {{\rm{I}}{{\rm{P}}_i}}} \times {{{\rm{I}}{{\rm{P}}_i}} \over {{\rm{IP}}}} \times {\rm{IP}}} \right)} = \sum\limits_{i = 1} {\left({{\rm{D}}{{\rm{I}}_i} \times S \times {\rm{IS}}} \right)} $$

The variables in Eq. (2) are defined in Table 1.

**Table 1:** Description of variables in decoupling equation.

Variable	Definition	Description of variable	Definition of related variable
*DI_i_*	${{{W_i}} \over {{\rm{I}}{{\rm{P}}_i}}}$	Discharge intensity of TI	*W_i_*: discharge volume of the wastewater or COD of the sub-sector
*S*	${{{\rm{I}}{{\rm{P}}_i}} \over {{\rm{IP}}}}$	Industrial structure of TI	*IP_i_*: output value of the sub-sector
*IS*	*IP*	Industry scale of TI	*IP*: output value of TI

**Note:**

TI, textile industry.

Therefore, the amount of change in discharge between two years Δ*W* can be expressed as follows:

(3) $$\Delta W{\rm{\,=\,}}\sum\limits_{i = 1} {\left({\Delta {W_{{\rm{D}}{{\rm{I}}_i}}}{\rm{ + }}\Delta {W_S}{\rm{ + }}\Delta {W_{{\rm{IS}}}}} \right)} {\rm{ \,=\, }}\Delta {W_{{\rm{DI}}}}{\rm{ + }}\Delta {W_S}{\rm{ + }}\Delta {W_{{\rm{IS}}}}$$

ΔW_DI_, ΔW_S_, and ΔW_IS_ represent the changes in water environmental stress caused by discharge intensity, industrial structure, and industrial scale, respectively. *T* is chosen as the time contrast argument. The logarithmic average weighting of each factor provides the following equations.

(4) $$\Delta {W_{{\rm{DI}}}}{\rm{ \,=\, }}\mathop \sum \limits_{i = 1} \Delta {\rm{D}}{{\rm{I}}_i} \,=\, \mathop \sum \limits_{i = 1} {{{W^T} - {W^{T - 1}}} \over {{\rm{ln}}{W^T} - {\rm{ln}}{W^{T - 1}}}} \times {\rm{ln}}{{{\rm{D}}{{\rm{I}}_i}^T} \over {{\rm{D}}{{\rm{I}}_i}^{T - 1}}}$$

(5) $$\Delta {W_S}{\rm{ \,=\, }}\mathop \sum \limits_{i = 1} \Delta {S_i}{\rm{ \,=\, }}\mathop \sum \limits_{i = 1} {{{W^T} - {W^{T - 1}}} \over {{\rm{ln}}{W^T} - {\rm{ln}}{W^{T - 1}}}} \times {\rm{ln}}{{{S_i}^T} \over {{S_i}^{T - 1}}}$$

(6) $$\Delta {W_{{\rm{IS}}}}{\rm{ \,=\, }}\mathop \sum \limits_{i = 1} \Delta {\rm{I}}{{\rm{S}}_i}{\rm{ \,=\, }}\mathop \sum \limits_{i = 1} {{{W^T} - {W^{T - 1}}} \over {{\rm{ln}}{W^T} - {\rm{ln}}{W^{T - 1}}}} \times {\rm{ln}}{{{\rm{I}}{{\rm{S}}_i}^T} \over {{\rm{I}}{{\rm{S}}_i}^{T - 1}}}$$

For year *T*, the contribution of the sub-sector *i* is calculated as follows.

(7) $$\Delta {W_{{\rm{D}}{{\rm{I}}_t}}}{\rm{=\, }}\Delta {\rm{D}}{{\rm{I}}_i}{\rm{ \,=\, }}{{{W^T} - {W^{T - 1}}} \over {{\rm{ln}}{W^T} - {\rm{ln}}{W^{T - 1}}}} \times {\rm{ln}}{{{\rm{D}}{{\rm{I}}_i}^T} \over {{\rm{D}}{{\rm{I}}_i}^{T - 1}}}$$

(8) $$\Delta {W_{{S_t}}}{\rm{ \,=\, }}\Delta {S_i}{\rm{ \,=\, }}{{{W^T} - {W^{T - 1}}} \over {{\rm{ln}}{W^T} - {\rm{ln}}{W^{T - 1}}}} \times {\rm{ln}}{{{S_i}^T} \over {{S_i}^{T - 1}}}$$

(9) $$\Delta {W_{{\rm{I}}{{\rm{S}}_t}}}{\rm{ \,=\, }}\Delta {\rm{I}}{{\rm{S}}_i}{\rm{ \,=\, }}{{{W^T} - {W^{T - 1}}} \over {{\rm{ln}}{W^T} - {\rm{ln}}{W^{T - 1}}}} \times {\rm{ln}}{{{\rm{I}}{{\rm{S}}_i}^T} \over {{\rm{I}}{{\rm{S}}_i}^{T - 1}}}$$

### Decrement and rebound effects

The principle of decrement requires less material, energy, or resource investment to achieve a desired target in production or consumption. In an ecological environment studying area, decrement effect refers to the total amount of environmental stress that declines while the economic output is maintained with improved environmental efficiency. In the decomposition of water environmental stress, the reduction in the water environmental stress brought by the discharge intensity factor and the industrial structure factor is the decrement effect (Δ*W*_RD_). The formula is expressed as follows.

(10) $$\Delta {W_{{\rm{RD}}}}{\rm{ = }}\Delta {W_{{\rm{IS}}}} - \Delta W\,{\rm{ = }}\,\Delta {W_{{\rm{IS}}}} - \left( {\Delta {W_{{\rm{DI}}}}{\rm{ + }}\Delta {W_S}\,{\rm{ + }}\,\Delta {W_{{\rm{IS}}}}} \right)\,{\rm{ = }}\, - \Delta {W_{{\rm{DI}}}} - \Delta {W_S}$$

The study of rebound effects originates from energy economics (Khazzoom, 1980); Vehmas, Luukkanen & Kaivo-Oja (2004) extended the rebound effect to the field of environment and suggested that the resulting improvements in ecological efficiency are offset by the increase in population and the richness of living brought by such ecological improvement; an increase in ecological efficiency aggravated the environmental damage. The study of the relationship between the ecological environment and economic development should consider the issue of sustainable development (Vehmas, Luukkanen & Kaivo-Oja, 2007) given that expanding an economic scale increases environmental stress and its total amount. The formula of rebound effect (Δ*W*_RB_) is expressed as follows. Δ*W*_RB_ and Δ*W* are equal in numerical value.

(11) $$\Delta {W_{{\rm{RB}}}}{\rm{ = }}\Delta {W_{{\rm{IS}}}}-{W_{{\rm{RD}}}}{\rm{ = }}\Delta {W_{{\rm{IS}}}}\,{\rm{ + }}\,\Delta {W_{{\rm{DI}}}}\,{\rm{ + }}\,\Delta {W_S}$$

### Data

According to China’s “Industrial Classification for National Economic Activities” (GB/T 4754-2017), the TI is divided into the manufacture of textile (MT), the manufacture of textile wearing and apparel (MTWA), and manufacture of chemical fibers (MCF) in this study. Therefore, the data from the TI’s industrial output value and the total wastewater discharge are the sum of the three sub-sectors. The National Bureau of Statistics of China and the Ministry of Environmental Protection of China provided the data. The sample analysis period is from 2001 to 2014. This paper uses the gross industrial product as the economic output value (the constant price in 2014) in the analytical model, and the total wastewater discharged is the wastewater discharge volume. Data from 2001 to 2005 are derived from the China Environment Yearbook (2002–2006) (Ministry of Environmental Protection of the People’s Republic of China, 1998–2006); the data are recorded in the China Environmental Statistical Annual Report (2006–2014) with the same statistical caliber (Ministry of Environmental Protection of the People’s Republic of China, 2006–2014).

## Results and Discussion

### Wastewater and COD discharge of TI and sub-sectors

**Figure 2:**
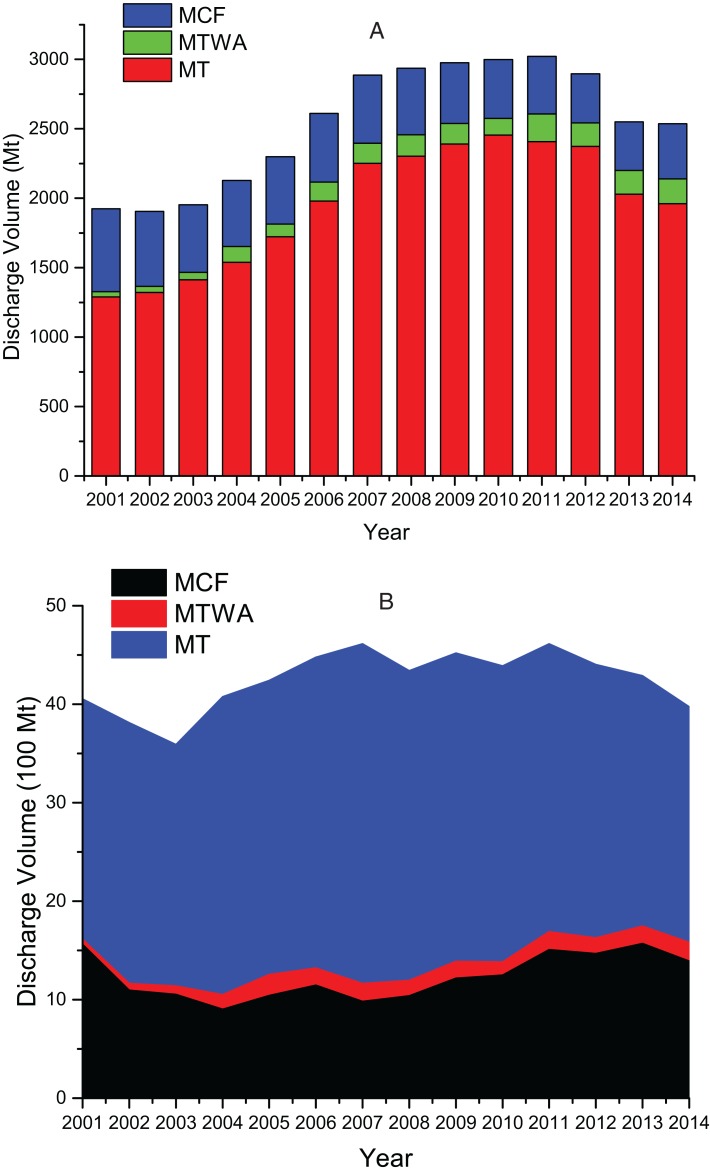
(A) The wastewater discharge of China’s TI and its three sub-sectors; (B) the COD discharge of China’s TI and its three sub-sectors.

From 2001 to 2014, China’s TI and sub-sectors’ total wastewater discharge are shown in Fig. 2A. The total amount of wastewater in China’s TI increases first and then decreases. An upward fluctuating trend was observed during 2001–2011, which increased from 1,923.93 Mt in 2001 to 3,021.08 Mt in 2011 with an average annual growth rate of 4.62%. Wastewater discharge decreased from 2012 to 2014, and the discharge volume was 2,537.68 Mt in 2014. MT’s wastewater discharge trends in the sub-sector are basically consistent with TI’s wastewater discharge trends. The total amount of wastewater discharged from MT is the largest, followed by that from MCF and MTWA. The total discharge of MT’s wastewater rose from 2001 to 2010. The total wastewater discharge from MT accounted for 64.04% in 2001 and 81.92% in 2012. MTWA’s wastewater was the smallest in proportion and variation amplitude. MCF’s wastewater discharge proportion was in the middle position among three sub-sectors, and its overall trend was declining, which accounted for the highest in 2014 (7.01%) and the lowest in 2001 (1.94%).

Figure 2B shows the COD discharge of China’s TI and sub-sectors during 2001–2014. The COD discharge can be divided into three stages. From 2001 to 2007, the COD discharge of the TI showed an upward trend as a whole, from 0.41 Mt in 2001 to 0.46 Mt in 2007, with an average annual growth rate of 2.17%. The TI’s COD discharge was in a volatile trend from 2007 to 2011. The COD discharge in 2011 was 0.46 Mt, which was flat with that amount in 2007. The COD discharge began to decline steadily since 2011. The discharged COD quantity was 0.40 Mt in 2014, which was reduced by 13.83% compared to 2011. MT is the major source of the TI’s COD discharge. From 2001 to 2007, MT’s COD discharge showed an upward trend. MT accounted for the largest COD discharge proportion in 2007 (74.78%) and the least proportion in 2013 (59.27%). MTWA has the least discharge volume and proportion of TI’s COD. The proportion was all between 5.02% (2005) and 1.30% (2001). MCF’s COD discharge proportion was between MT and MTWA, whose proportion was highest in 2001 (38.51%) and lowest in 2007 (21.32%).

### Decoupling wastewater and COD discharge from the output value of TI

The decoupling results of water environmental stress and the economic growth in the TI from 2002 to 2014 are shown in Table 2. For the decoupling relations between wastewater discharge and economic growth, good overall decoupling status was observed, in which weak decoupling accounted for nine years (2003–2011), strong decoupling for three years (2002, 2013–2014), and recessive decoupling for one year (2012). The decoupling status behaves even better for COD, with six years for weak decoupling (2004–2007, 2009, 2011), six years for strong decoupling (2002–2003, 2008, 2010, 2013–2014), and one year for recessive decoupling (2012).

**Table 2:** Decoupling results of the TI during 2002–2014.

Year	*D*_WD_	Decoupling degree	*D*_COD_	Decoupling degree
2002	−0.134	Strong decoupling	−0.838	Strong decoupling
2003	0.331	Weak decoupling	−0.759	Strong decoupling
2004	0.476	Weak decoupling	0.727	Weak decoupling
2005	0.354	Weak decoupling	0.175	Weak decoupling
2006	0.510	Weak decoupling	0.209	Weak decoupling
2007	0.558	Weak decoupling	0.161	Weak decoupling
2008	0.043	Weak decoupling	−0.145	Strong decoupling
2009	0.214	Weak decoupling	0.648	Weak decoupling
2010	0.059	Weak decoupling	−0.234	Strong decoupling
2011	0.029	Weak decoupling	0.202	Weak decoupling
2012	1.337	Recessive decoupling	1.478	Recessive decoupling
2013	−2.465	Strong decoupling	−0.538	Strong decoupling
2014	−0.666	Strong decoupling	−9.709	Strong decoupling

**Note:**

D_WD_: decoupling elasticity of wastewater discharge and economic growth; D_COD_: decoupling elasticity of COD content and economic growth.

The decoupling state of China’s TI was in good condition as a whole from 2002 to 2014 because China promulgated environmental binding policies that promote the change in TI’s economic growth mode. Textile enterprises invested abundant financial, manpower, and material resources for environmental protection equipment and traditional production technology transformation, and investment in advanced technology and equipment increased. Furthermore, the industry technical level greatly improved through the upgrade of equipment manufacturing technology and the introduction of international advanced technology and equipment.

China’s State Council issued the “The notice to issue the 12th Five-Year Plan of environmental protection” in December 2011 (China’s MIIT [2011] No. 42) (Central People’s Government of the People’s Republic of China, 2011). The plan put forward new requirements for pollutant emission in high-polluting industries and raised adjustment strategies to speed up the elimination of backward production capacity. Faced with increasingly stringent environmental requirements, many textile enterprises failed to meet production capacity standards, and they were ordered to stop production or shut their business down. The requirements impacted the TI’s production capacity, and recessive decoupling occurred in both wastewater and COD in 2012.

The TI’s economic growth and water environmental stress in 2013–2014 showed a strong decoupling state, indicating a well implementation in China’s environmental policy. The State Council of China issued “The implementation of the most stringent water resources management system and assessment methods” in 2013 (China’s MIIT [2013] No. 2) (The Central People’s Government of the People’s Republic of China, 2013) to strengthen the red line management of the water function area’s limited pollution and strictly control the total amount of sewage into rivers and lakes. Therefore, in 2013, the TI’s wastewater discharge and COD were 2,550.43 Mt and 0.43 Mt, respectively, which was reduced by 124.79 and 0.11 Mt, respectively, from 2012. Thus, the emission reduction effect is remarkable. The economic development of the TI increased, and the water environmental per unit of output decreased. Thus, the decoupling effect was evident. The TI’s water environmental stress in 2014 continued to decline, wastewater discharge and COD discharge were reduced 0.50% and 7.29%, respectively, compared with that in 2013. The reduction effect in COD is more significant.

On the whole, the decoupling state between discharged COD and economic growth is holding better than the decoupling state between wastewater discharge and economic growth. In order to control COD discharge and reduce water environmental stress, the state has set COD discharge restriction standards and they have achieved great effect. In contrast, the decoupling of wastewater discharge has not reached a desired decoupling state. A large amount of wastewater discharge shows that the water consumption is large and the reuse technology in wastewater is not advanced. The in-depth analysis of the influencing factors of wastewater discharge is of great significance for reducing wastewater discharge.

### Decoupling wastewater discharge from the output value of three sub-sectors

The results of the decoupling elasticity of the three sub-sectors of China’s TI from 2002 to 2014 are shown in Tables 3–5.

**Table 3:** Decoupling results of the MT during 2002–2014.

Year	%Δ*W*_WD_	%Δ*P*	*D*_WD_	Decoupling degree
2002	0.025	0.017	1.448	Expansive negative decoupling
2003	0.077	0.121	0.565	Weak decoupling
2004	0.089	0.207	0.432	Weak decoupling
2005	0.119	0.242	0.494	Weak decoupling
2006	0.149	0.240	0.621	Weak decoupling
2007	0.138	0.108	1.278	Expansive negative decoupling
2008	0.023	0.596	0.039	Weak decoupling
2009	0.038	0.094	0.402	Weak decoupling
2010	0.027	0.082	0.323	Weak decoupling
2011	−0.019	0.243	−0.078	Strong decoupling
2012	−0.015	−0.033	0.442	Weak negative decoupling
2013	−0.145	0.051	−2.840	Strong decoupling
2014	−0.033	−0.028	1.173	Recessive decoupling

**Note:**

%ΔW_WD_: the growth rate of wastewater discharge; %ΔP: the growth rate of output value; D_WD_: decoupling elasticity of wastewater discharge and economic growth.

**Table 4:** Decoupling results of the MTWA during 2002–2014.

Year	%Δ*W*_WD_	%Δ*P*	*D*_WD_	Decoupling degree
2002	0.179	0.204	0.881	Expansive coupling
2003	0.199	0.152	1.308	Expansive negative decoupling
2004	1.164	0.108	10.825	Expansive negative decoupling
2005	−0.194	0.127	−1.522	Strong decoupling
2006	0.490	0.771	0.636	Weak decoupling
2007	0.059	−0.004	−15.787	Strong negative decoupling
2008	0.052	0.075	0.689	Weak decoupling
2009	−0.034	0.148	−0.229	Strong decoupling
2010	−0.183	−0.104	1.748	Recessive decoupling
2011	0.651	0.378	1.721	Expansive negative decoupling
2012	−0.141	0.104	−1.360	Strong decoupling
2013	0.003	0.064	0.055	Weak decoupling
2014	0.038	0.117	0.323	Weak decoupling

**Note:**

%ΔW_WD_: the growth rate of wastewater discharge; %ΔP: the growth rate of output value; D_WD_: decoupling elasticity of wastewater discharge and economic growth.

**Table 5:** Decoupling results of the MCF during 2002–2014.

Year	%Δ*W*_WD_	%Δ*P*	*D*_WD_	Decoupling degree
2002	−0.096	0.192	−0.500	Strong decoupling
2003	−0.095	−0.058	1.626	Recessive decoupling
2004	−0.028	0.156	−0.182	Strong decoupling
2005	0.022	0.224	0.099	Weak decoupling
2006	0.021	0.188	0.113	Weak decoupling
2007	−0.012	0.549	−0.022	Strong decoupling
2008	−0.018	0.063	−0.283	Strong decoupling
2009	−0.088	−0.072	1.225	Recessive decoupling
2010	−0.034	0.387	−0.087	Strong decoupling
2011	−0.022	0.258	−0.086	Strong decoupling
2012	−0.148	−0.060	2.478	Recessive decoupling
2013	−0.008	0.035	−0.235	Strong decoupling
2014	0.138	0.093	1.487	Expansive negative decoupling

**Note:**

%ΔW_WD_: the growth rate of wastewater discharge; %ΔP: the growth rate of output value; D_WD_: decoupling elasticity of wastewater discharge and economic growth.

Table 3 indicates that MT’s decoupling state is unstable during the study years and that weak negative decoupling occurred in 2012. MT showed expansive negative decoupling in 2002 and 2007, strong decoupling in 2011 and 2013, and recessive decoupling in 2014. The remaining nine years were for weak decoupling (2003–2006, 2008–2010). From 2002 to 2011, enterprises and entrepreneurs enhanced their environmental protection awareness. National environmental policies were remarkable tightened, along with a zero emission system on the export of textiles and raw materials. Moreover, customers’ needs of environmental healthy textile products increased. All these are the main reasons underlying the good decoupling during this period. In 2012, “Textile Dyeing and Finishing Industrial Water Pollutant Discharge Standard” (GB4287-2012) was issued by China’s Ministry of Environmental Protection, which requires textile printing and dyeing enterprises’ direct discharge of COD concentration dropped to 100 mg/L or less, and the indirect emissions dropped down to 200 mg/L or less (previously 500 mg/L). Enterprises and industrial parks during this period must heavily invest in building sewage treatment plants or improving the processing capacity of existing wastewater treatment facilities, which resulted in wastes of manpower, material, land, and funds. The majority of China’s textile printing and dyeing enterprises are small- and medium-sized; these enterprises lacked the technology and funds, and their pretreatment facilities barely met the emission requirements, making some enterprises’ bankruptcy and decline in total production (Ma, Wang & Li, 2017). The worldwide financial crisis has led to a shrinking of demand in the textile market, which would have an impact on the export of China’s textile products. Highly stringent environmental protection measures further diminished total textile production in 2014. For instance, Zhejiang Province, where nearly 60% of China’s textile printing and dyeing production capacities are located, carried out “A total of five water treatment” action, which mainly caused recessive decoupling (Ministry of Environmental Protection of the People’s Republic of China, 2014).

Table 4 shows that, except for 2003–2004, 2007, and 2011, MTWA’s growth rate of wastewater is less than that of its output value. MTWA showed expansive negative decoupling in 2003, 2004, and 2011; strong decoupling in 2005, 2009, and 2012; expansive coupling in 2002; strong negative decoupling in 2007; recessive decoupling in 2010; and weak decoupling in the remaining four years (2006, 2008, and 2013–2014). Compared with the other two sub-sectors, the main reason for the bad decoupling condition of MTWA is that its production process determines that the consumption of water resources is not high; thus, wastewater discharge is relatively minimal. A large part of MTWA’s wastewater discharge was from the working water of labor and the process water of basic equipment. China’s accession to the WTO from 2001 to 2005 contributed to the increase in MTWA’s scale of exports. MTWA’s labor-intensive characteristics require considerable labor for production activities, which is the main reason for the increase in wastewater emissions. The rapid growth of MTWA and foreign export trade (Ministry of Industry and Information Technology of the People’s Republic of China, 2016a) from 2006 to 2014 increased wastewater discharge. However, MTWA’s wastewater discharge during this period changed minimally because the Chinese MTWA experienced an evolution process from the original equipment manufacture to the original design manufacture and the original brand manufacture. This increased level of technology inhibited wastewater emissions.

Table 5 shows MCF’s recessive decoupling in 2003, 2009, and 2012; weak decoupling in 2005 and 2006; and expansive negative decoupling in 2014. The remaining seven years were strong decoupling (2002, 2004, 2007–2008, 2010–2011, 2013). MCF achieved more years with strong decoupling state compared with the other two sub-sectors. However, this ideal decoupling state is not continuous but alternative, which indicates that the state is unstable. During the “11th Five-Year” period (2006–2010), MCF eliminated more than 3 million tons of backward production capacity, and water consumption per ton and wastewater discharge decreased by 25.7% and 25%, respectively, compared with those in 2005; the production of chemical fiber in 2010 was 30.9 million tons, which accounted for over 60% of the world’s total production (Ministry of Industry and Information Technology of the People’s Republic of China, 2012). The “Adjustment and revitalization plan of TI” (Central People’s Government of the People’s Republic of China, 2009) put forward new requirements on the TI’s industrial structure and wastewater discharge from 2009 to 2011; the plan proposed to accelerate the elimination of backward production capacity of MCF and high demand forced MCF to optimize industrial capacity and technology innovation. The decoupling status of MCF fluctuated during the “12th Five-Year Plan” period (2011–2015). This fluctuation is primarily attributed to the constraints on resources and environment that brought challenges, and the contradictions of inner structural and among products, scale, and application that remained prominent (Ministry of Industry and Information Technology of the People’s Republic of China, 2017).

### Analysis of factors influencing wastewater discharge

#### Decomposition analysis of the wastewater discharge in TI

**Figure 3:**
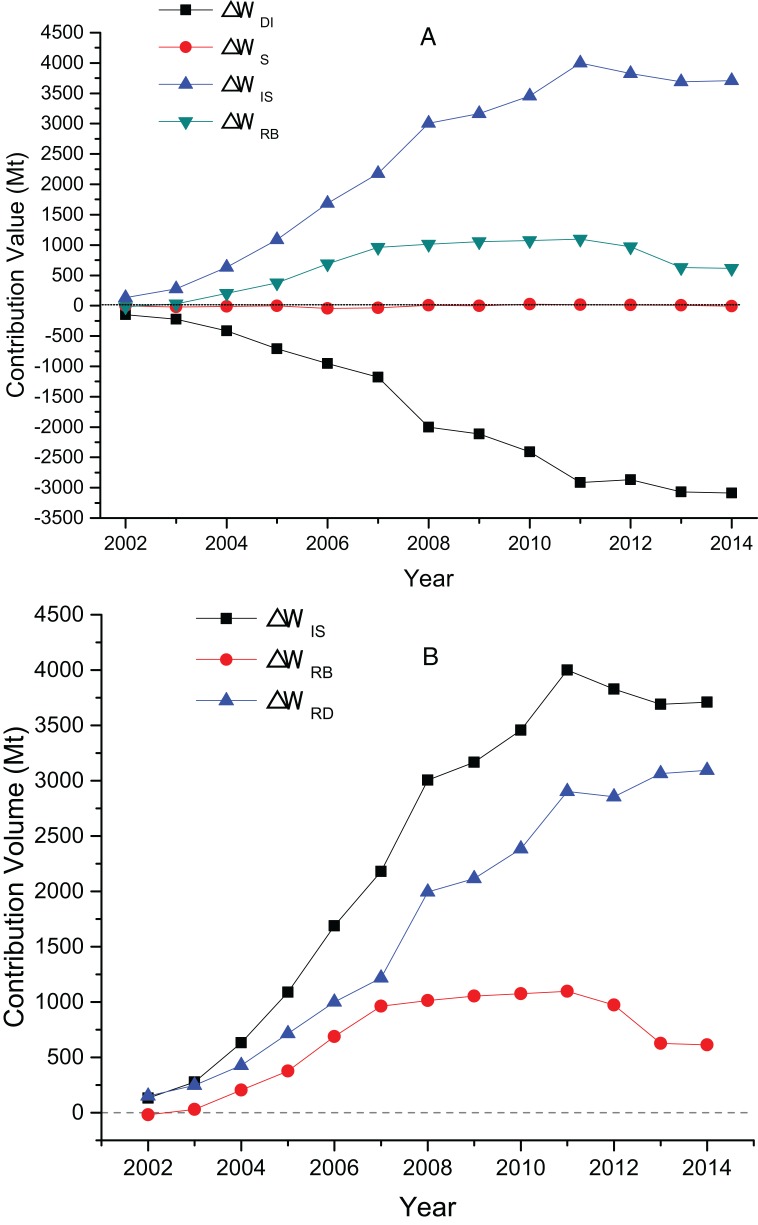
(A) The contribution value of factors influencing TI’s wastewater discharge; (B) the contribution value of TI’s decrement effect and rebound effect.

The decomposition contribution results and the factors’ effect figure of China’s TI wastewater discharge from 2002 to 2014 are shown in Fig. 3. A positive value indicates that the factor has a positive effect on wastewater discharge, which means that the factor promotes wastewater discharge. A negative value means that the factor has a negative correlation to wastewater discharge, that is, wastewater discharge is suppressed.

Figure 3A show that wastewater discharge intensity factor is the most important factor inhibiting the increase of wastewater discharge. In 13 years, the contribution of intensity factors are negative, and the average contribution value of −1,699.37 Mt is a comprehensive reflection of various waste reduction measures, such as pollution control technology, clean production, and production equipment. This value shows that intensity factors are negatively correlated to the change in wastewater discharge. Accelerating the upgrade of emission reduction technology and the promotion of clean production can reduce wastewater discharge in the TI. Compared with the other two factors, the fluctuation of the industrial structure is larger, but the influence is minimal, and its promotion or inhibition effect is insignificant. Overall development trend shows that the structure factor plays a promotion and inhibition roles before 2007 and after 2008, respectively. The numerical value shows that the influence of TI’s structure factors on the changes in wastewater discharge is small, and the average contribution value is only −5.71 Mt, which is far less than that of the other two factors. The industrial structure adjustment of China’s TI is insignificant in reducing its wastewater discharge. Industry scale factor is the most important factor to drive wastewater from the TI. The average contribution value is 2,373.61 Mt, and the total contribution value is 30,856.90 Mt. In 2001–2014, China’s TI output value showed an upward trend, which increased from USD 37.25 billion to USD 27.43 trillion in 2014.

Figure 3B present that TI’s decrement effect continued to increase from 150.51 Mt in 2002 to 3,095.03 Mt in 2014, with an average annual growth rate of 28.65%. The rebound effect increased first (2002–2011) and then declined (2012–2014), with the lowest at −18.39 Mt in 2002 and the highest at 1,097.15 Mt in 2011. The existence of rebound effect shows that the total amount of wastewater discharged from the TI is still increasing. It is mainly due to the reason that as the technology improves, on the one hand, wastewater treatment technology is highly promoted and wastewater discharge decreases; on the other hand, technology progress makes the productivity and consumption to raise, which in turn brings an increase in economic output and wastewater discharge. When the increase in discharge due to the expansion of economies offset the decrement effect brought by the improvement of technology, the rebound effect occurs.

#### Decomposition analysis of wastewater discharge in three sub-sectors

**Figure 4:**
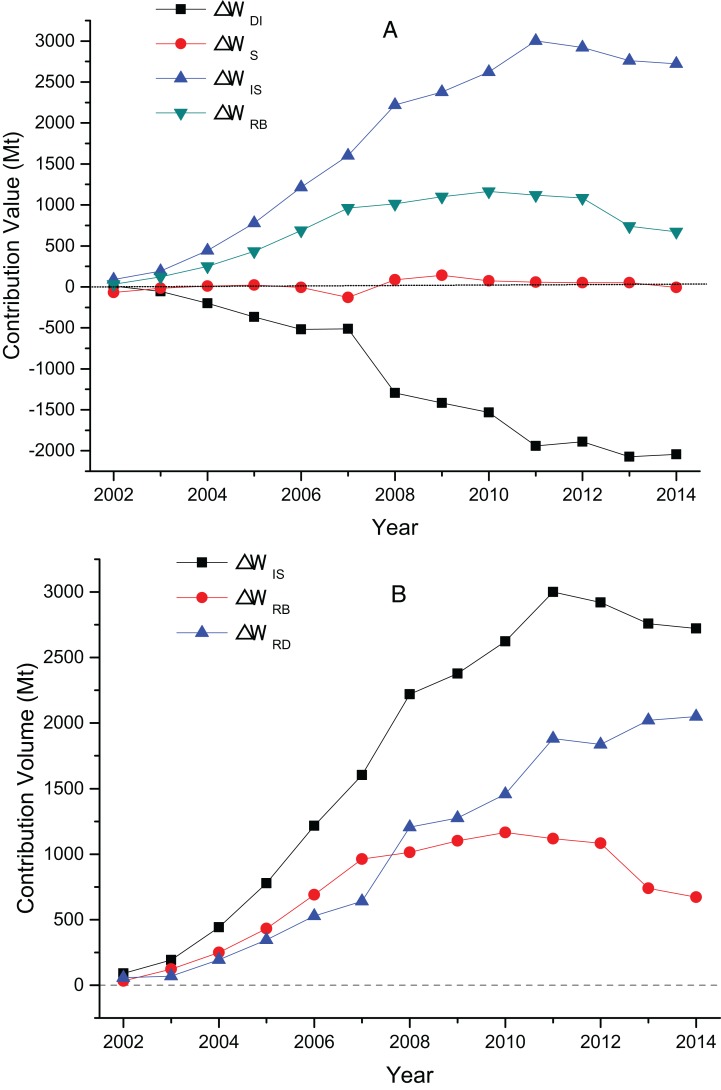
(A) The contribution value of factors influencing MT’s wastewater discharge; (B) the contribution value of MT’s decrement effect and rebound effect.

**Figure 5:**
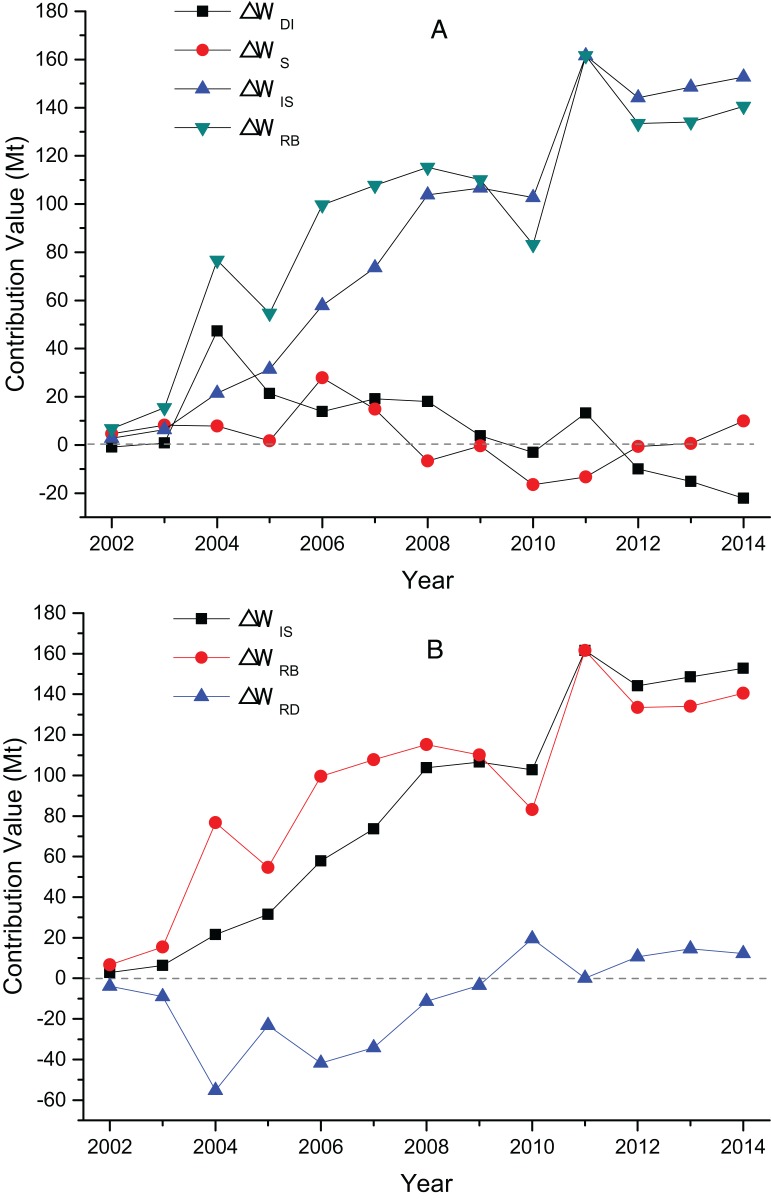
(A) The contribution value of factors influencing MTWA’s wastewater discharge; (B) the contribution value of MTWA’s decrement effect and rebound effect.

**Figure 6:**
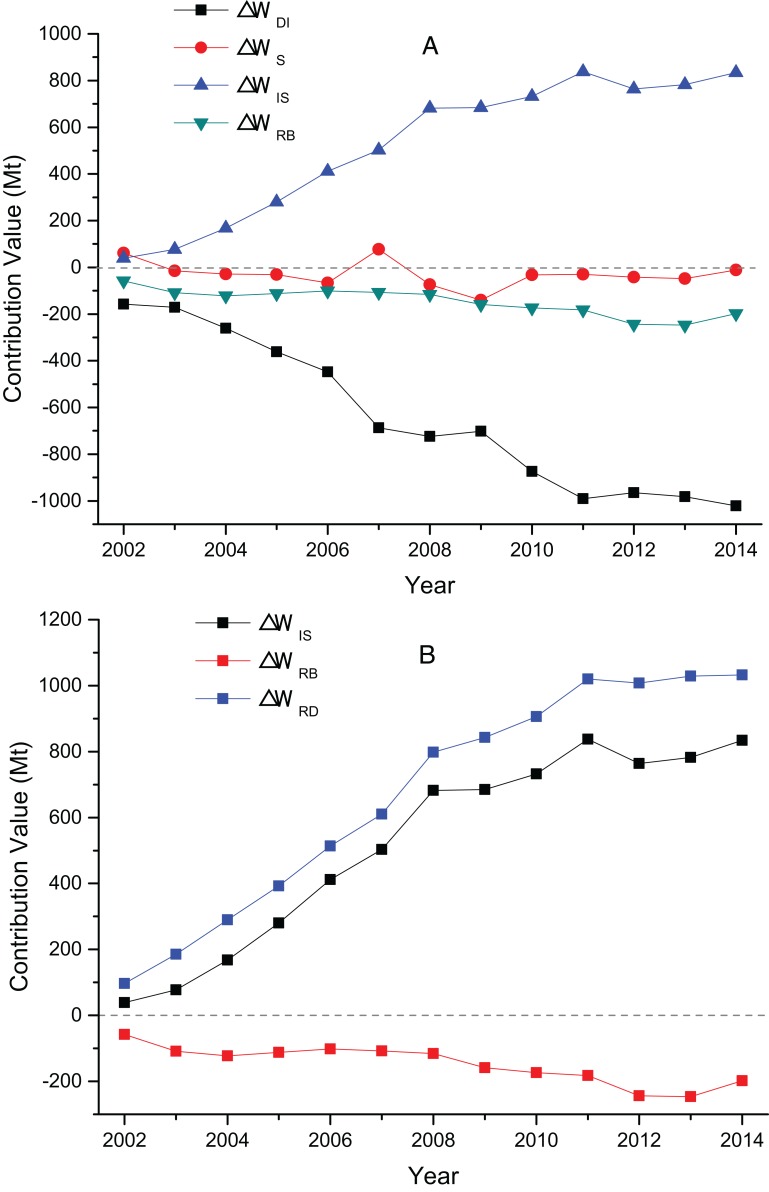
(A) The contribution value of factors influencing MCF’s wastewater discharge; (B) the contribution value of MCF’s decrement effect and rebound effect.

The decomposition results and factor effects of the three sub-sectors’ wastewater discharge from 2002 to 2014 are shown in Figs. 4–6.

Figure 4A illustrates that the contribution value of MT’s intensity factor is negative except in 2002, and the average contribution value is −1,064.17 Mt. An increasing trend is shown with the years of growth. The contribution value reached −2,043.34 Mt in 2014, which indicates discharge intensity factor as the main inhibitor of wastewater discharge. The contribution value of MT’s structure factor is significantly less than that of the other two factors; thus, its impact is small. The scale factor contributes positive values to wastewater discharge, with a large average contribution value of 1,765.19 Mt, and it is the main driving factor of wastewater discharge. Figure 4B illustrates that the decrement effect of the TI shows an overall trend of growth from 57.79 Mt in 2002 to 2,050.15 Mt in 2014, with an average annual growth rate of 34.63%. The rebound effect rose in 2002–2010 from 32.34 to 1,164.96 Mt but began to decline from 1,118.28 Mt in 2011 to 671.71 Mt in 2014.

Figure 5A shows that the intensity and structure factors of MTWA are small in 2002–2014, and the discharge of wastewater is mainly driven by the scale factor. The scale factor rises as a whole from 2.79 Mt in 2002 to 152.73 Mt in 2014, with an average annual growth rate of 39.59%. Figure 5B presents that the decrement effect of MTWA is not evident, with a minimum value of −55.17 Mt in 2004 and a maximum value of 19.54 Mt in 2010. The rebound effect is mainly controlled by the scale effect; the former fluctuated in 2002–2014 and increased from 6.68 Mt in 2002 to 140.53 Mt in 2014.

The contribution values of MCF’s intensity factor are all negative, with an overall contribution of −641.86 Mt during the study period. Thus, intensity factor is the most important factor that restricts the wastewater discharge of MCF. The structure factor has minimal contribution, and its impact on wastewater discharge is not evident. Scale factor shows an upward trend in the sample years, from 39.19 Mt in 2002 to 834.19 Mt in 2014, with an average annual growth rate of 29.03%. This factor is the main incentive for stimulating wastewater discharge. Figure 6B shows that the decrement effect of MCF increases from 96.60 Mt in 2002 to 1,032.68 Mt in 2014, with an average annual growth rate of 21.83%. MCF’s rebound effect is negative, the maximum value is −57.41 Mt in 2002, and the minimum value is −246.77 Mt in 2013. MCF contributes the most to the decrement effect of TI, and it has the least rebound effect among the three sub-sectors.

## Conclusion

The decoupling status of economic growth and water environmental stress in the TI and its three sub-sectors are evaluated based on the total wastewater and COD discharge data and the economic output value of China’s TI and its three sub-sectors from 2001 to 2014. The factors that affect wastewater discharge are then analyzed. The main conclusions are as follows:

1. The overall decoupling state between economic growth and water environmental stress of China’s TI is in good condition. Between 2002 and 2014, wastewater discharge has three years (2002, 2013, and 2014) for strong decoupling, nine years (2003–2011) for weak decoupling, and one year (2012) for recessive decoupling. For the COD discharge, there are six years in strong decoupling (2002, 2003, 2008, 2010, 2013, 2014), six years in weak decoupling (2004–2007, 2009, 2011), and one year (2012) for recessive decoupling. The decoupling status behaves well mainly due to the implementation of the national discharge reduction policy, the progress of production technology, and the elimination of backward production capacity in that the water environmental stress problem of China’s TI greatly improved. It indicates that control in COD discharge behaves better and a further analysis in wastewater discharge is needed. Extending the discussion of wastewater discharge into three sub-sectors’ decoupling state, the decoupling level of the MCF is the highest for seven years (2002, 2004, 2007, 2008, 2010, 2011, and 2013), which reaches a strong decoupling state in 2002–2014. The MT shows an overall weak decoupling state and has two years (2011 and 2013) for strong decoupling, which takes the second decoupling level. The manufacture of the MTWA has three years (2005, 2009, and 2012) of strong decoupling and four years (2006, 2008, 2013, and 2014) of weak decoupling. MTWA’s overall decoupling situation is at the last of the three sub-sectors but has a significantly lower wastewater discharge than the other two sub-sectors.
2. The decoupling state of China’s TI resulted from a combination effect of influencing factors. The discharge intensity factor of wastewater discharge is the main factor curbing the wastewater discharge of TI, with an average contribution value is −1,699.37 Mt. The scale factor is the biggest pulling cause of TI’s wastewater discharge, with an average contribution value of 2,373.61 Mt. The contribution of structure factor is smaller than the other two factors. Thus, it has less influence on wastewater discharge. In the three sub-sectors, MT contributes most to the wastewater discharge of China’s TI, followed by MCF and MTWA. The discharge intensity factor inhibits the wastewater discharge of MT and MCF but has little effect on MTWA. The structure factor’s impact on the wastewater discharge of the three sub-sectors remains small. The scale factor stimulates the total discharge of three sub-sectors’ wastewater.
3. The decrement effect is remarkable in the TI’s wastewater discharge, while the rebound exists at the same time. For China’s TI, on the whole, TI’s decrement effect increases annually, but the rebound effect shows that the absolute amount of wastewater discharge also increases. The rebound effect has shown a downward trend since 2012. In the three sub-sectors, MT’s decrement effect increases yearly, and its rebound effect shows that the absolute amount of wastewater discharge increases too. The rebound effect has shown a downward trend since 2010. MTWA’s decrement effect is negative in 2001–2009, and the value becomes positive after 2010. MTWA has a strong rebound effect and shows an upward trend. MCF’s decrement effect is the strongest among the three sub-sectors, and its rebound effect is the weakest, which indicates that MCF is the biggest contributor to the discharge reduction of China’s TI.

The research above reveals the need to consider the multiple effects of intensity, structure, and scale in order to realize a win–win situation of economic growth and decreased water environmental stress in the TI. First, we must accelerate the development and promotion of recycling and energy-saving emission reduction technology and encourage enterprises to increase technological transformation and upgrade their clean production capacity. Second, we must deepen the adjustment of industrial structure, accelerate the extensive mode of economic development in the TI, and develop a modern TI system with green environmental protection, high added value, and high-end service industry. Third, we must improve the level of technical equipment in the industry, eliminate backward production capacity, promote TI transform from labor-intensive to technology-intensive, and carry forward the supply side reform of the TI.

## Supplemental Information

10.7717/peerj.5112/supp-1 Supplemental Information 1 Discharge volume of wastewater and COD, output value.

## Additional Information and Declarations

### Data Availability

The following information was supplied regarding data availability: The raw data are provided in a Supplemental File.
